# Physicochemical Characterization and Antimicrobial Properties of Lanthanide Nitrates in Dilute Aqueous Solutions

**DOI:** 10.3390/molecules29174023

**Published:** 2024-08-25

**Authors:** Galina Kuz’micheva, Alexander Trigub, Alexander Rogachev, Andrey Dorokhov, Elena Domoroshchina

**Affiliations:** 1 Research and Educational Center “Multi-scale Materials Engineering”, MIREA—Russian Technological University, 119454 Moscow, Russia; galina_kuzmicheva@list.ru; 2 National Research Center “Kurchatov Institute”, 123182 Moscow, Russia; trigub_al@nrcki.ru (A.T.);; 3 Lomonosov Institute of Fine Chemical Technologies, Department of Inorganic Chemistry Named after A.N. Reformatsky, MIREA—Russian Technological University, 119454 Moscow, Russia; anddor@mail.ru

**Keywords:** *Ln* nitrate solutions, X-ray diffraction, X-ray absorption spectroscopy, IR spectroscopy, antimicrobial activity

## Abstract

This work presents the results of studying dilute aqueous solutions of commercial *Ln*(NO_3_)_3_ · *x*H_2_O salts with *Ln* = Ce-Lu using X-ray diffraction (XRD), IR spectroscopy, X-ray absorption spectroscopy (XAS: EXAFS/XANES), and pH measurements. As a reference point, XRD and XAS measurements for characterized *Ln*(NO_3_)_3_ · *x*H_2_O microcrystalline powder samples were performed. The local structure of *Ln*-nitrate complexes in 20 mM *Ln*(NO_3_)_3_ · *x*H_2_O aqueous solution was studied under total external reflection conditions and EXAFS geometry was applied to obtain high-quality EXAFS data for solutions with low concentrations of *Ln*^3+^ ions. Results obtained by EXAFS spectroscopy showed significant contraction of the first coordination sphere during the dissolution process for metal ions located in the middle of the lanthanide series. It was established that in *Ln*(NO_3_)_3_ · *x*H_2_O solutions with *Ln* = Ce, Sm, Gd, Yb (c = 134, 100, 50 and 20 mM) there are coordinated and, to a greater extent, non-coordinated nitrate groups with bidentate and predominantly monodentate bonds with *Ln* ions, the number of which increases upon transition from cerium to ytterbium. For the first time, the antibacterial and antifungal activity of *Ln*(NO_3_)_3_ · *x*H_2_O *Ln* = Ce, Sm, Gd, Tb, Yb solutions with different concentrations and pH was presented. Cross-relationships between the concentration of solutions and antimicrobial activity with the type of *Ln* = Ce, Sm, Gd, Tb, Yb were established, as well as the absence of biocidal properties of solutions with a concentration of 20 mM, except for *Ln* = Yb. The important role of experimental conditions in obtaining and interpreting the results was noted.

## 1. Introduction

Traditionally, the coordination chemistry of lanthanides has been a matter of exceptional interest for wide ranging industrial applications, primarily for development of effective methods for separation and purification of rare-earth elements [1]. The efficiency of the extraction process is greatly dependent on solvation properties of lanthanide ions, such as complexation with extractants, solubility of salts, dissociation/association of ion pairs, etc. The optimization of industrial technologies for extraction of rare-earth elements, as well as elaboration of novel “green” methods for their recycling and recovering, requires deeper insight into molecular mechanisms regulating metal–ligand interaction in aqueous/organic media. *Ln*(NO_3_)_3_ · *x*H_2_O salts are excellent precursors for production of ultra-high purity compounds, and certain catalyst and nanoscale materials [2]. In addition, *Ln*(NO_3_)_3_ · *x*H_2_O is used in the growth of large-sized polyfunctional crystals doped with *Ln*^3+^ ions from aqueous solutions [3].

According to the World Health Organization (WHO), infectious diseases are spreading faster and emerging more quickly than ever before. Due to the increasing incidents related to new and reemerging infectious diseases, the discovery and development of new antimicrobial compounds with diverse structures and action mechanism is urgently needed. The antibacterial effects of lanthanides (*Ln*) have been studied since the 19th century [4] and have been employed since then with more or less success in the treatment of various diseases. Recently much interest in the aqueous chemistry of lanthanides has been stimulated by the growing implications of these elements in medicine and biotechnologies [5]. Cerium nitrate solutions are commonly recognized as the topical agent of choice for the treatment of burn wounds [6,7] and exhibit superior antibacterial activity [8]. In particular, Ce(NO_3_)_3_ · 6H_2_O salt solutions (~50 mM) are used as an antiseptic agent for the treatment of indwelling medical devices (implants, catheters) [7]. 

The antimicrobial properties of *Ln* nitrate solutions are presented, practically, only in one work [8] for Ce^3+^(NO_3_)_3_ · 6H_2_O (c = 133 mM, pH = 4.2), which suggests a connection between the growth inhibition zone (D, mm) of *S. aureus*, *E. coli*, and *P. aeruginosa* bacteria with the formal charge of Ce^3+^ and with the solution concentration. The value of D, mm of bacteria in the presence of Ce^3+^(NO_3_)_3_ ·6H_2_O solution, is much higher than that on the penicillin antibiotic with a wide spectrum of action [8]. It is not excluded that the absence of interaction of Ce^3+^ ions in an aqueous solution of Ce^3+^(NO_3_)_3_ ·6H_2_O salt [8] and Yb^3+^ ions in Yb^3+^(NO_3_)_3_ ·*x*H_2_O solution (c = 0.1 mM, pH = 6.5) [9] with Langmuir phospholipid monolayers of phosphatidylethanolamine (DPPE) and phosphatidylglycerol (DPPG) is associated with damage to the protein, rather than the phospholipid component of the bacterial membrane and/or with the “penetration” of Ce^3+^ and Yb^3+^ ions into the cell and disruption of its water–ion balance. Antifungal properties of *Ln*(NO_3_)_3_ ·*x*H_2_O solutions are not available in the literature. In general, lanthanide compounds are now considered to be a potential alternative to antibiotics in antimicrobial and antifungal therapy [5]. 

Investigations into the hydration behavior of lanthanides in diluted solutions are of special interest for biological sciences, when low *Ln*^3+^ concentrations are used to reduce possible cytotoxic effects. It is also important to bear in mind that the coordination environment of *Ln*^3+^ ions is strongly dependent on lanthanide salt concentration and can differ significantly in dilute aqueous solutions compared to more concentrated ones [10]. Thus, increasing attention in lanthanide biochemistry has been paid to experimental techniques that provide atomic-level information on the solution structure of *Ln*^3+^ ions under diluted conditions [11]. On the other hand, while maintaining the functional properties of dilute solutions, a decrease in the content of *Ln*^3+^ ions is ensured when drugs are taken orally. 

The techniques for studying the nature (size and/or structure) of the *Ln* shell in solutions can be classified as direct or indirect methods [12]. The direct methods include X-ray [13,14] and neutron diffraction [15], X-ray absorption spectroscopy (EXAFS) [16,17] FT-IR [18,19] and Raman spectroscopy [19,20], and nuclear magnetic resonance (NMR) [21]. The indirect methods involve compressibility, NMR exchange, and adsorption spectroscopy measurements [21]. In addition, ab initio quantum mechanical calculations [22] and molecular dynamics (MD) are applied using the classical or mixed quantum/classical interaction potential, which are combined with UV-visible spectroscopy [23] or X-ray absorption spectroscopy (XAS), or a combination of MD simulations and XAS spectroscopy [24]. Experimental, highly sensitive microcalorimetry is used to calculate the thermodynamic characteristics of weak interactions in solutions [25] together with luminescence emission spectra and the lifetime of *Ln* [26]. X-ray absorption spectroscopy has been proven to be exceptionally informative in investigating the coordination environment of *Ln*^3+^ ions in aqueous solutions [11,16,24,27,28,29,30,31]. Most X-ray absorption spectroscopy studies reported in the literature are focused on the solutions of *Ln*^3+^ chloride salts. Their results cannot be transferred to solutions of other salts, nor the approaches used.

The purpose of this work is to identify the structural features of dilute aqueous solutions of lanthanide nitrates and to establish the role of the concentration and pH of the medium in the implementation of antimicrobial properties.

To characterize aqueous solutions of *Ln*(NO_3_)_3_ · *x*H_2_O (*Ln* = Ce-Lu), modern informative methods of X-ray diffraction (XRD), infrared spectroscopy (FT-IR), and X-ray absorption spectroscopy (XAS) were chosen. In the present study we used the main advantage of X-ray absorption spectroscopy under total external reflection (TER), namely that the extremely low intensity of background scattering and accordingly high signal-to-noise ratio allows for the detection of week spectroscopic signals from samples with small amounts of absorbing atoms. X-ray absorption spectroscopy measurements under TER was performed for 20 mM *Ln*(NO_3_)_3_ · *x*H_2_O aqueous solutions across the lanthanide series from Ce to Lu. In addition, an X-ray standing wave (XRSW) technique was applied to examine the adsorption behavior of *Ln*^3+^ ions at the air/liquid interface.

## 2. Results and Discussion

The local structure of *Ln*^3+^ nitrate complexes in salt and dilute aqueous solution was systematically studied across the lanthanide series from Ce to Lu.

### 2.1. X-ray Standing Wave Studies 

Additional XRSW measurements were carried out to examine the adsorption behavior of *Ln*^3+^ ions at the solution surface, in particular the possible enrichment of *Ln*^3+^ ions at the air/liquid interface. In XRSW experiments, the intensity of characteristic fluorescence exited by the incident X-ray beam is recorded as a function of the incident angle θ. The key idea of the XRSW method is as follows: the angular dependence of fluorescence yield is highly sensitive to the position of atoms in the direction normal to the sample surface. Thus, XRSW measurements offer an opportunity to locate the atoms directly from the analysis of the corresponding fluorescence curve [32].

**Figure 1:**
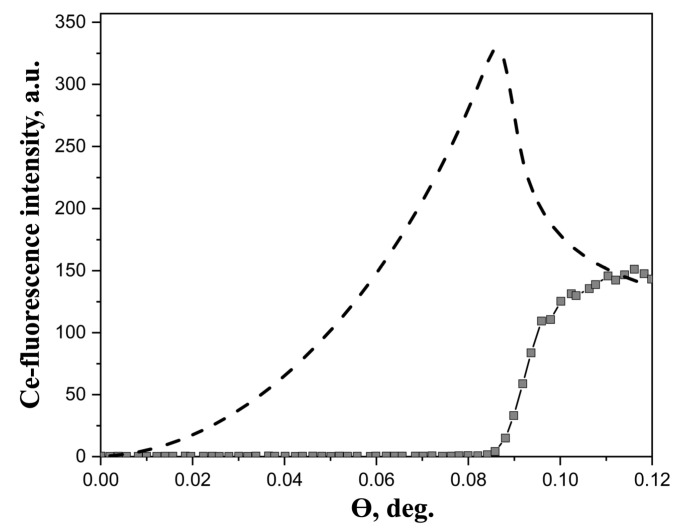
Experimental angular dependence of Ce-fluorescence yield from 20 mM aqueous solution of Ce(NO_3_)_3_ · 6H_2_O salts (squares). The dashed line represents the calculated angular dependence of the film, in which metal ions are distributed in a layer 10 Å thick at the air/liquid interface. The energy of the incident beam was 13.6 keV.

Experimental XRSW data, obtained for studied *Ln*^3+^ aqueous solutions, exhibited essentially similar behavior. As a typical example, Figure 1 shows angular dependence of Ce L3-fluorescence from Ce(NO_3_)_3_ · 6H_2_O salt solution. 

Generally, two factors should be kept in mind when analyzing XRSW data collected under TER. The first factor is the dramatic changes in electric field intensity above the reflecting surface, as the incidence angle is scanned within the TER region. As a direct result of these changes, large modulations arise in angular dependence of fluorescence yield from atoms located in the near-surface region; calculated angular dependence, presented in Figure 1, is a perfect illustration of this characteristic feature. The calculations have been performed using the recursion formalism developed by Parratt [33]. As can be seen in Figure 1, angular dependence of fluorescence yield for near-surface distribution of atoms drastically increases from zero at θ = 0° and reaches the maximum value in the vicinity of the critical angle θ_C_.

In marked contrast, the angular dependence of fluorescence yield from the atoms, which are present in the bulk liquid subphase, exhibits different behavior. In this case, the most important factor is the changes in the penetration depth of the electric field into the subphase. As the incident angle increases above the critical angle θ_C_, the penetration depth abruptly rises from several nanometers (for θ < θ_C_) to several hundreds of microns (for θ > θ_C_), which in turn results in the sharp increase in fluorescence signal. It can be seen from Figure 1 that the Ce fluorescence curve recorded in our experiments corresponds to this type of angular dependence. These observations evidence that no enhancement of *Ln*^3+^ concentration in the near-surface region occurs in the dilute aqueous solutions of *Ln*(NO_3_)_3_ · *x*H_2_O salts and the distribution of *Ln*^3+^ ions can be considered as homogeneous.

### 2.2. X-ray Absorption Spectroscopy Studies

As a reference point in studying *Ln*(NO_3_)_3_ · *x*H_2_O aqueous solutions (c = 20 mM), we performed X-ray absorption spectroscopy measurements also for *Ln*(NO_3_)_3_ · *x*H_2_O microcrystalline powder samples (Table S1). For appropriate fitting of the first intense peak in a Fourier-transformed spectrum, one should take into account two coordination shells around the metal ion: oxygen atoms (in crystalline *Ln*(NO_3_)_3_ · *x*H_2_O: CN*Ln* = 4O_H2O_ + 6O_NO3_ except for CNCe = 11–5O_H2O_ + 6O_NO3_, CNYb(Lu) = 9–3O_H2O_ + 6O_NO3_; *Ln* bond is monodentate with water oxygen and bidentate with oxygen of nitrate groups), nitrogen atoms (in crystalline *Ln*(NO_3_)_3_ · *L*H_2_O coordination number is 3: CN*Ln* = 3N_NO3_) [34,35,36,37,38,39,40,41,42,43,44]. The results of such fitting are presented in Table S1 and in Figure S1.

These fits are performed with fixed coordination numbers. Such fitting makes it possible to evaluate the capabilities of the EXAFS method for determining structural parameters. The observation results presented in Table S1 demonstrate that only the first coordination shell radius could be determined with appropriate precision. 

**Figure 2:**
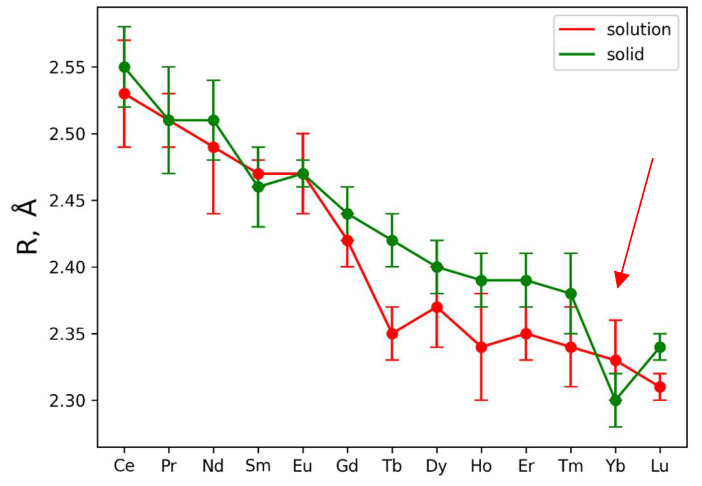
Dependence of *Ln*-O distance (R, Å) from central atom type. Red and green curves are used to plot data for solutions and solids, respectively. The red arrow indicates a “break” in the interatomic distances at Yb.

This analysis was then applied to the experimental EXAFS data for solutions. However, in the case of solutions, coordination numbers for the oxygen and nitrogen coordination shells were considered as free parameters, and the Debye–Waller parameter for the nitrogen coordination shell was fixed to reduce the number of independent parameters during fitting. The results are also presented in Table S1 and in Figure S2. In the case of solutions, the only parameter which could be determined from EXAFS fitting with appropriate precision is an interatomic distance *Ln*-O for the first coordination shell. Figure 2 contains the *Ln*-O bond distances (R, Å) over the *Ln* series.

The plotted error bars associated with each data point are based on EXAFS fitting uncertainty. It is clearly seen from the presented *Ln*-O distances that *Ln*-O for *Ln* = Tb, Dy, Ho, Er, Tm ions demonstrate significant contraction of the oxygen shell in solutions (Figure 2). Such observation is also in agreement with measured XANES spectra, which are plotted in Figure S5. Vertical lines in Figure S5 show the position of the second maximum in XANES spectra; shifting these lines towards higher energies indicates shrinking of the first coordination shell [45].

Concluding the results obtained by the examination of XANES spectra and the fitting of the EXAFS ones, contraction of the first coordination shell for the metals *Ln* = Tb, Dy, Ho, Er, Tm is observed. Stability of the nitrate complexes could not be determined by a simple EXAFS fitting procedure.

It should be noted that the nitrogen coordination number in aquatic solutions has been a subject of disagreement. Questions such as whether an eventual coordination change occurs within each series or whether inner or outer sphere complexation occurs for specific ligands have not been completely resolved. Thus, according to [25], the “light” lanthanides in aqueous solutions are nine-coordinated, whereas the “heavy” ones are eight-coordinated. According to [46], La-Nd has larger coordination numbers than Tb-Lu, and in the region between them (*Ln* = Sm-Gd) there are transitional structures or a mixture of structures. The authors of [47] believe that the coordination numbers change in the Nd-Dy region. It is interesting to note that the authors of the review [48] do not exclude fractional coordination (between 8 and 9) *Ln* = Gd-Ho in *Ln*(NO_3_) solutions. According to [49], the exact coordination numbers of rare-earth ions in the nitrate complexes of solutions are unknown. The latter statement seems to be correct, since CN*Ln* in aqueous solutions depends on the concentration: in highly concentrated solutions, coordination with fewer solvent molecules can be realized than in dilute ones, and in significantly dilute solutions, even more so due to the shortage of water molecules.

Analysis of Figure 2 and Table S1 data allows for the distinguishing of two *Ln*(NO_3_)_3_ · *x*H_2_O regions: *Ln* = Ce-Eu (region 1) where the Ln-O interatomic distances in solid and liquid phases are almost identical, and *Ln* = Gd-Lu (region 2) where the interatomic distances in solid phases are larger compared to liquid ones (excluding experimental errors; the trend was analyzed) with a clear separation of the *Ln*-O distances with *Ln* = Tb and Lu (taking into account experimental errors) and a “break” in the interatomic distances at Yb (indicated by the red arrow in Figure 2). Moreover, it is not excluded that in region 1 of *Ln*(NO_3_)_3_ · *x*H_2_O solutions, both bidentate and monodentate bonds of *Ln* with nitrate groups are realized, and in region 2 only monodentate bonds occur.

According to Raman spectroscopy data [50], in a wide range of Ce(NO_3_)_3_ concentrations in aqueous solution, nitrate ions are bound to Ce both monodentately and bidentately, which is consistent with our data (Table S1, Figure 2). Complexation of Nd^3+^ and Eu^3+^ ions with the given nitrate group was studied by spectrophotometry and microcalorimetry [26]. The authors are of the opinion that both inner-sphere and outer-sphere nitrate complexes of *Ln*^3+^ ions exist in solutions. Based on thermodynamic and spectroscopic data, it is assumed that the weak complex of Nd^3+^ with nitrate in solution forms an inner sphere, and the nature of complex formation increases with an increase in temperature. In addition, it is possible that nitrate binds Eu^3+^ and possibly also Nd^3+^ bidentately in aqueous solutions.

Bonal S. et al. [25] used microcalorimetry to determine the stability constant, Gibbs energy, enthalpies, and entropies to analyze the very weak complexation of *Ln* = La-Lu nitrate anion in dilute aqueous solutions of *Ln*(NO_3_)_3_ · *x*H_2_O at room temperature (298.15 K), with an analysis of changes in the thermodynamic properties of (*Ln*NO_3_)^2+^ across the lanthanide series. With a decrease in the ionic radius of *Ln*^3+^, it is more difficult for the nitrate anion with a slightly larger size in the NO_3_ group than a water molecule to “penetrate” into the inner sphere of the *Ln*^3+^ cation. Therefore, for Tm-Lu, the inner sphere consists exclusively of water molecules, which was confirmed by Raman measurements performed on aqueous solutions of rare-earth nitrates in the liquid state at room temperature and in the glassy state at liquid nitrogen temperature [49]. Moreover, as the cation becomes smaller, the preference for monodentate nitrate binding increases, due to avoided repulsions in the first coordination sphere. These literature data do not contradict our analysis of EXAFS results (Table S1, Figure 2). 

Dobler et al. [22] used a quantum chemical study to show that an increase in the number of water molecules in the first coordination sphere of *Ln*^3^ promotes monodentate coordination of nitrate. The change from bidentate to monodentate coordination is also observed before the salt dissociation; i.e., in an aqueous solution, nitrates on the way to dissociation pass from bidentate to monodentate coordination. The thermodynamic explanation for this process is interesting. In solution, the enthalpy (ΔH) and entropy (ΔS) energy components are antagonists. Bidentate binding may be promoted by entropy, which reduces the number of “frozen” water molecules coordinated with *Ln*^3+^ ions. On the other hand, monodentate bonding may be preferable from an enthalpy point of view, since up to six hydrogen bonds can be formed with the oxygens of the three monodentate nitrates (O_NO3_) instead of three with the bidentate nitrate groups. If we compare the course of interatomic distances (Table S1, Figure 2) according to EXAFS data with the curves ΔS of complexes (*Ln*NO_3_)^2+^ and ΔH from *Ln* [22], then we can detect similarities for *Ln* = Ce-Eu with the ΔS curve. This may be an indirect confirmation of the presence of a certain amount of nitrate groups bidentately bound to *Ln* = Ce-Eu. 

The authors of [23] used MD with explicit polarization and UV-visible spectroscopy to study solutions of Nd^3+^ and Dy^3+^ nitrates from a “highly diluted” solution to experimental saturation. It has been established that the bidentate mode is somewhat more stable for Nd^3+^ than for Dy^3+^; at the end of the lanthanide series, the ratio between the bidentate and monodentate conformations decreases, while only the monodentate mode is present in Lu.

Moreover, in solution, hydrated nitrate complexes can exhibit an equilibrium between several polyhydrate forms involving different types of nitrate binding modes [51].

Analysis of the limited literature data on the study of dilute solutions of *Ln*(NO_3_)_3_ · *x*H_2_O salts confirms the different structural behavior of *Ln*^3+^ ions in them, and different results are observed depending on the calculation methods and experimental conditions. It should be noted that experimental concentrations are typically far from standard and cannot be correctly extrapolated to infinite dilution to obtain a thermodynamic equilibrium constant that is valid only for a particular medium and concentration range (ionic strength).

### 2.3. X-ray Diffraction

According to the structural analysis of *Ln*(NO_3_)_3_ · *x*H_2_O salts [34,35,36,37,38,39,40,41,42,43,44], with an increase in the *Ln* atomic number, the total content of water molecules decreases: *x*= 6 for Ce-Sm, *x* = 6 and 5 for Eu-Tb, *x* = 5 for Dy-Yb (except for Tm with x = 6 and 5), and *x* = 4 and 3 for Lu. In the inner sphere (in square brackets) of coordination compounds of the form [*Ln*(O_2_NO_3_)_3_(OH_2_)*_n_*] · (*x* − *n*)H_2_O, the number of water molecules also decreases along the *Ln* series (*n* = 5 for Ce, *n* = 4 for Pr-Yb, *n* = 3 for Lu) connected monodentantly to *Ln*. In the outer sphere (outside square brackets), the number of water molecules (*x* − *n*) for all *Ln* except Ce, and the content of (NO_3_)^1−^ groups bidentantly coordinated with *Ln* ions remains constant (3 NO_3_).

It should be noted that in the *Ln*(NO_3_)_3_ · 6H_2_O structures, starting from Gd, the length of one *Ln*-O_NO3_ bond is greatly increased compared to others (bond asymmetry: Δ = 0.202 Å). The bond asymmetry increases in the structure with Tb (Δ = 0.220 Å) and is at its maximum (Δ = 0.523 Å) in the structure with Tm [35]. For example, in the Tm(NO_3_)_3_ · 6H_2_O [35], Tm ions are bound to six oxygen ions from three nitrate groups (Tm has two bonds with oxygen–bidentate coordination): an asymmetric coordination with one shorter (2.4039 (17)Å – 2.4677 (17)Å) bond and one longer Tm—O distance (2.5034 (18) Å, 2.5252 (18)Å, 2.991 (2)Å each) (Δ, Å value is the difference between the largest short distance and the largest long distance). In accordance with the fact that the corresponding Tm—O distance (2.991 Å) is even larger than the distance between the Tm atom and the central N atoms of the two remaining anions, the coordination arrangement of Tm should be described as CN Tm = 8 (3O_H2O_ + 2O_NO3_ + 2O_NO3_ + 1O_NO3_) for the inner sphere and CN Tm = 9 + 1 (instead of CN Tm = 10) taking into account the outer sphere [35]. This asymmetric bonding seems to be associated with a steric effect of the coordinating water molecules. The increasing asymmetry in the binding mode of one nitrate group is related to the decrease in the ionic radius of the *Ln*.

**Figure 3:**
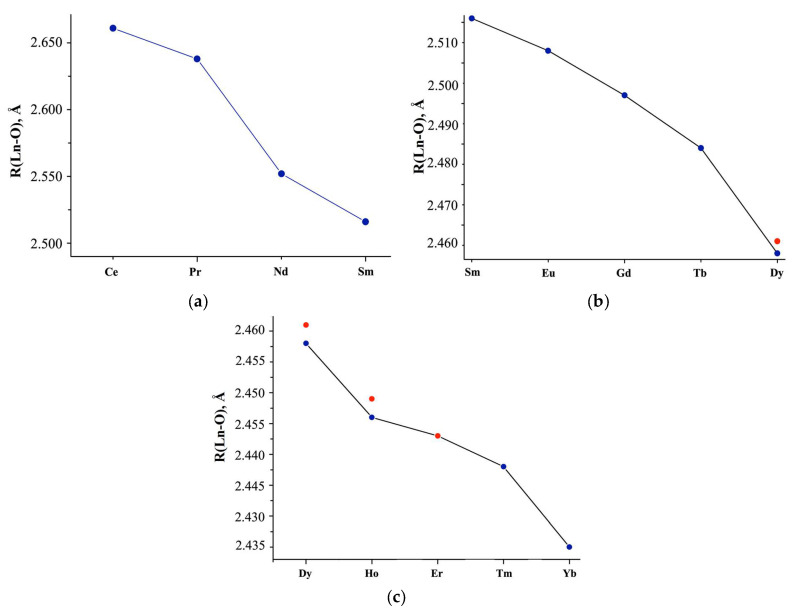
(**a**) Average *Ln*-O interatomic distances (R, Å) in the structures of *Ln*(NO_3_)_3_ · *x*H_2_O salts, according to literature data highlighting the (**b**) Sm-Dy, (**c**) Dy-Yb regions. Red dots: interatomic distances calculated from phase analysis of the commercial samples we studied.

An analysis of the interatomic distances given in the literature showed that for the same *Ln*, for which x = 5 and x = 6 in the *Ln*(NO_3_)_3_ · *x*H_2_O composition (*Ln* = Eu-Tb, Tm), with an increase in molecules of crystallization water, the average *Ln*-O_H2O_ distance decreases (Δ*_Ln_*_-O(H2O)_~0.032 Å is the difference between the average interatomic distances *Ln*-O_H2O_ in *Ln*(NO_3_)_3_ · *x*H_2_O structures with x = 5 and x = 6), and *Ln*-O_NO3_ (Δ*_Ln_*_-O(NO3)_ is the difference between the average interatomic distances *Ln*-O_NO3_ in *Ln*(NO_3_)_3_ · *x*H_2_O structures with x = 5 and x = 6), on the contrary, increases, reaching a maximum value (Δ*_Ln-_*_O(NO3)_ =0.058 Å) for Tm [35]. In this case, the average *Ln*-O interatomic distance in the first coordination sphere increases upon transition from x = 5 to x = 6 since Δ*_Ln-_*_O(NO3)_ > Δ*_Ln_*_-O(H2O)_. For Lu(NO_3_)_3_ · *x*H_2_O in the transition from x = 3 to x = 4, the value of Δ*_Ln_*_-O(NO3)_ =0.004 Å is very small, which contributes to an increase in *Ln*-O (*R*,Å) in the Lu(NO_3_)_3_ · 3H_2_O structure compared to Lu(NO_3_)_3_ · 4H_2_O (Table S2, Figure 3).

The X-ray diffraction study of commercial *Ln*(NO_3_)_3_ · *x*H_2_O samples [52] indicates single-phase *Ln*(NO_3_)_3_ · 6H_2_O with Ce (structure 1; x = 6), Pr-Tb (structure 2; x = 6), Tm (structure 3; x = 5), two-phase with Dy-Er (structure 2 + structure 3), Yb (structure 3 + unknown structure 4), and uncertainty with Lu (unknown structure 5 or non-single-phase sample). Although the crystal structures of 1–3 differ from each other (the closest are structures 2 and 3), and the structures of 4 and 5 are unknown, their main structural fragments are the same.

Table S2 and Figure 3a,b show the average interatomic distances *Ln*-O (R, Å) without separation into *Ln*-O_H2O_ and *Ln*-O_NO3_, calculated based on the *Ln* coordination in the inner sphere, taking into account the quantitative analysis of non-single-phase *Ln*(NO_3_)_3_ · *x*H_2_O with Ln = Dy, Ho, Er (Figure 3a–c; red dots). The structural parameters are consistent with the data given in [22] for *Ln*(NO_3_)_3_ · *x*H_2_O with *Ln* = Ce-Sm (*x* = 6; CN = 11 for Ce, CN = 10 for Nd-Sm), but differ for Eu (*x* = 6 according to our data, *x* = 5 according to [22]) and interatomic Lu-O distances (Figure 3a, blue double dots), which we either calculated or took from structural data [42].

**Figure 4:**
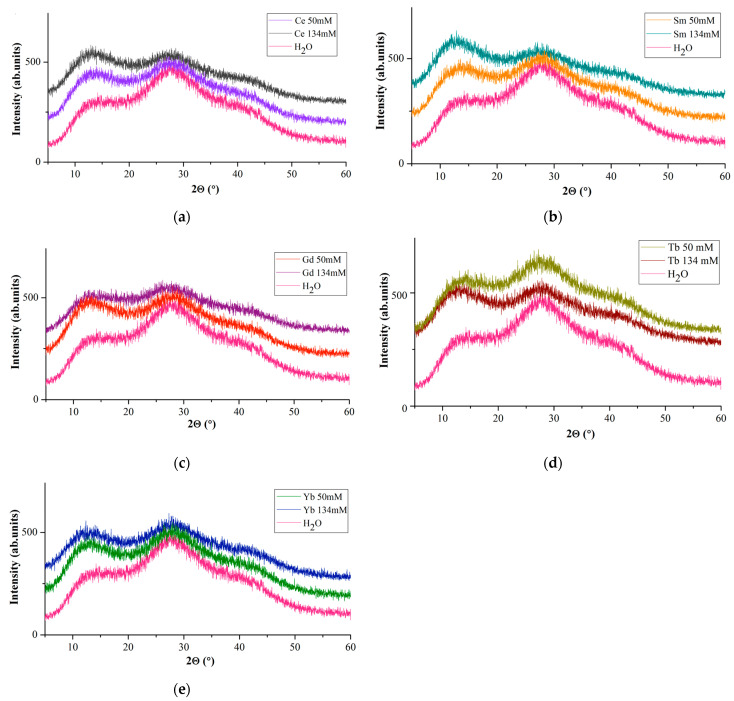
Diffraction patterns of water and solutions with c = 50 mM and c = 134 mM: *Ln*(NO_3_)_3_ · *x*H_2_O with (**a**) *Ln* = Ce, (**b**) Sm, (**c**) Gd, (**d**) Tb, (**e**) Yb.

X-ray diffraction patterns of *Ln*(NO_3_)_3_ · *x*H_2_O solutions (Figure 4) are represented by two pronounced halos with interplanar distances in the intervals *d* = ~7.16–~6.34 Å (first peak; 2θ~13°) and *d* = 3.423–3.119 Å (second peak; 2θ~28°) and diffuse peak with a maximum at *d*~2.15 Å (third peak; 2θ~42°).

A comparison of diffuse reflections of *Ln*(NO_3_)_3_ · *x*H_2_O solutions with *Ln* = Ce, Sm, Gd, Tb, Yb (Figure 4) with similar reflections of a dilute aqueous solution of lanthanum nitrate [53] shows their similarity. The main diffuse peaks for water (Figure 4, first and second peaks) occur at 14.17 and 27.92°(2θ) (*d* = 6.24 and 3.193 Å), along with a very weak and diffuse peak at ~39.85°(2θ) (*d*~2.26 Å) (Figure 4, third peak), and are present in the diffraction patterns of all solutions. It occupies an intermediate position between van der Waals molecules and molecular formations with a covalent chemical bond, and the components of the cluster retain their specific individuality.

If we take the intensities of the first (*I*(1)_rel_) and second (*I*(2)_rel_) diffuse reflections for water as a reference, then on the diffraction patterns of *Ln*(NO_3_)_3_ · *x*H_2_O solutions at *c* = 134 mM *I*(1)_rel_ > *I*(2)_rel_ for *Ln* = Ce, Sm (for Sm to a greater extent); for Gd, the intensities of the first and second diffuse reflections are most pronounced at c = 50 mM; for Yb, at c = 50 and 134 mM, the values of *I*(1)_rel_ and *I*(2)_rel_ are almost the same (Figure 4). The diffraction patterns of Tb(NO_3_)_3_ · *x*H_2_O solutions (Figure 4d) are strikingly different from the diffraction patterns of *Ln*(NO_3_)_3_ · *x*H_2_O solutions with *Ln* = Ce, Sm, Gd, Yb (Figure 4a–c): *I*(2)_rel_ > *I*(1)_rel_ at c= 50 mM; *I*(1)_rel_ is more pronounced than *I*(2)_rel_ at c = 134 mM (Figure 4d). Different diffraction patterns are characteristic of *Ln*(NO_3_)_3_ · *x*H_2_O solutions (*Ln* = Ce, Sm, Gd, Tb, Yb) of commercial samples that we studied and may indicate different hydrolysis processes depending on the *Ln* type. 

When comparing diffraction patterns of aqueous solutions [48,53,54] and *Ln*(NO_3_)_3_ · *x*H_2_O salts [34,36,37,42], the position of the first diffuse peak generally coincides with the region of the most intense Bragg reflections in the range of 2θ~12÷~15°, in particular, 1$\bar{1}$0 with *d*~6.7 Å, caused by *Ln* ions. These values are in the regions of increased electron density observed in the radial distribution functions of aqueous solutions. 

Modern ideas about the structure of lanthanide nitrate hydrates in the solid phase and aqueous solutions are considered in the review [55], where, based on quantum-chemical calculations and experimental data, for *Ln* = Pr-Yb, five types of structural isomers were established for the [*Ln*(NO_3_)_3_(H_2_O)_4_] complexes, with different mutual arrangements of nitrato- and aqualigands, and for *Ln* = Lu, two types of isomers were found for the [*Ln*(NO_3_)_3_(H_2_O)_3_] complexes. Let us pay attention to the difference in Lu-O interatomic distances in the salt and Lu(NO_3_)_3_ solution according to EXAFS data (Figure 2).

In dilute aqueous solutions (c < 10 mM), *Ln*^3+^ ions are in the structure in the form of aqua ions [Ln(H_2_O)*_n_*]^3+^, and with an increase in the concentration of the salt solution, as well as with an increase in the concentration of nitrate anions in the solution, water molecules in the internal coordination sphere of *Ln*^3+^ cations are replaced by bidentate nitrate anions. In our case, solutions with c = 20, 50, 100, 134 mM were considered, which, in accordance with the results of work [55], does not exclude the presence of bidentate nitrate anions, at least for *Ln* = Ce-Eu (region 1) in Figure 2 (EXAFS data).

### 2.4. FT-IR Spectroscopy Data

**Figure 5:**
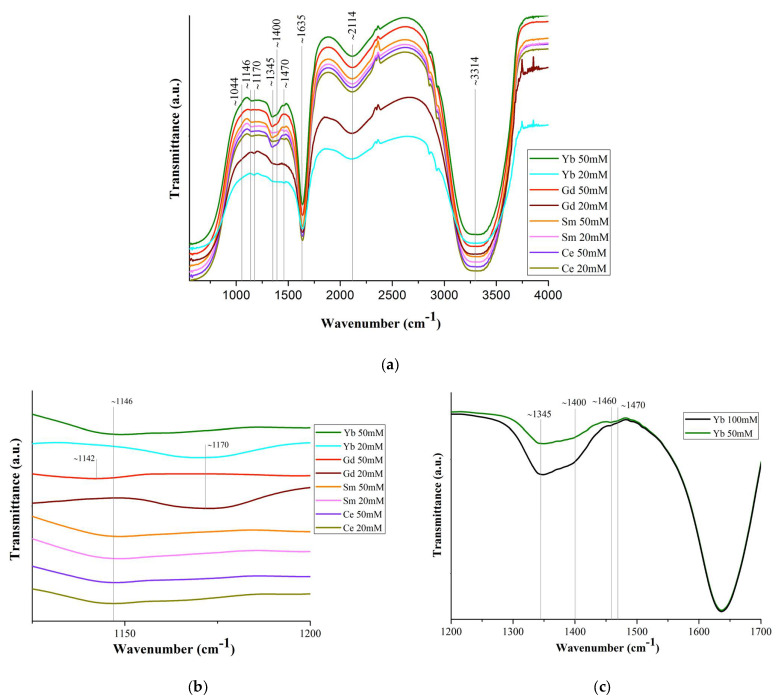

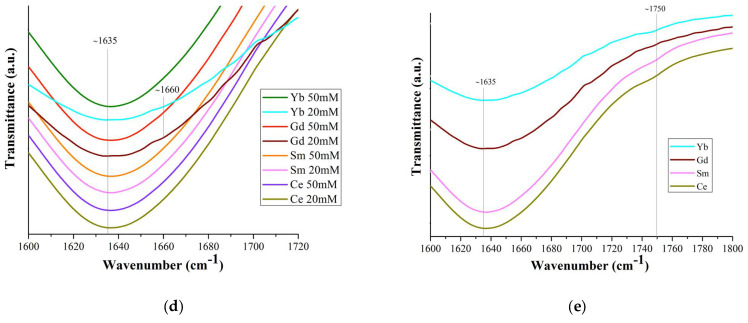
(**a**) FT-IR spectra of *Ln*(NO_3_)_3_ · *x*H_2_O solutions (*Ln* = Ce, Sm, Gd, Yb); (**c**) part of the spectrum (1200–1500 cm^−1^) of Yb(NO_3_)_3_ · xH_2_O solution (c = 50 mM and 100 mM); ((**b**,**d**,**e**) for c = 20 mM) spectrum sections.

Figure 5a shows the IR spectra of solutions of commercial *Ln*(NO_3_)_3_ · *x*H_2_O salts (c = 20 mM and 50 mM) with *Ln* = Ce, Sm, Gd, Yb, and the bandwidth correspondence presented in Table S3.

The band shift ~1044 cm^−1^ is associated with the bond covalency degree of nitrate ions with *Ln* ones [56], which increases along the *Ln* series. Based upon this shift in the 1050 cm^−1^ region, the following series may be set up for decreasing covalency of the metal–nitrate bond: Gd > Ce [57]. This should lead to a general tendency for interatomic distances and coordination numbers to decrease due to a decrease in the size of *Ln*, which is confirmed by the EXAFS data in Figure 2. A very weak band at 1146 cm^−1^ (Figure 5b) may correspond to bending vibrations of the hydronium ion δ (H_3_O^+^), which can be present in a solution with pH < 7 [58]. 

The splitting of the ~1400 cm^−1^ band into two (~1345 and ~1400 cm^−1^) (Figure 5c) indicates an asymmetric vibration of the uncoordinated hydrated nitrate ion. The difference (Δυ~125 cm^−1^) between the bands ~1345 and ~1470 cm^−1^ indicates partial coordination of the nitrate ions to *Ln* [57]. With an increase in the concentration of the *Ln*(NO_3_)_3_ · *x*H_2_O solutions (from 20 mM to 100 mM), the ~1470 cm^−1^ band becomes more clearly defined (Figure 5c). This means an increase in the number of bound nitrate ions and their gradual dominance over water molecules in competition to enter the inner coordination sphere. The band at 1470 cm^−1^ is quite wide and, in accordance with [17], apparently results from the superposition of bands of monodentate and bidentate coordinated nitrate ions [59,60]. 

The strong band at ~1635 cm^−1^ is the result of the superposition of water scissoring bending and N=O stretching: the vibrational peak for N=O stretching of bidentate coordinated nitrate ions usually appears at 1630–1788 cm^−1^ (Figure 5d). All bands in the 1625–1524 cm^−1^ region characterize isolated N=O bonds (consistent with the bidentate structure of nitrates) and the bands below 1520 cm^−1^ are coupled vibrations, consistent with monodentate (but not only) nitrate structures [61].

The weak band in the region of ~1750 cm^−1^ (Figure 5a,e) corresponds to the combination band of the symmetrical stretch and in-plane bending of the nitrate ion. With an increase in the *Ln* atomic number, the position of this band shifts (Figure 5a,e) to the long-wavelength region (1745 cm^−1^ for Ce, 1750 cm^−1^ for Sm, 1752 cm^−1^ for Yb; it was not possible to detect it for Gd), due to a weakening of the O–N bond in the nitrate ligand owing to an increase in electron transfer to the *Ln*^3+^ metal ion with an increase in the charge density.

A wide weak band at ~2114 cm^−1^ (Figure 5a) corresponds to the composite vibration of water molecules: bending vibration, together with stretching and intermolecular vibration due to the rotation of the water molecule. The band at 2130 cm^−1^ corresponds to the combination of oscillation ν_2_ + ν_L_: deformation together with libration. The libration oscillations of the water molecule are intermolecular and are associated with the molecule rotation [62]. The broad strong band ~3314 cm^−1^ corresponds to the stretching of free, hydrogen-bonded and coordinated water molecules. The band is greatly broadened, which indicates the implementation of strong hydrogen bonding in *Ln*(NO_3_)_3_ · *x*H_2_O solutions (Figure 5a). 

The wide intensive band at 2700–3700 cm^−1^ corresponds to three oscillations of the water molecule: asymmetric stretching oscillation (3490 cm^−1^), symmetric stretching oscillation (3280 cm^−1^), and the overtone of the bending oscillation (3250 cm^−1^). The intensity of the maximum of the bending band, on the contrary, decreases with a reduction of the temperature in the same range [63]. 

A wide intense band below ~800 cm^−1^, responsible for stretching vibrations of water molecules, overlaps the bands of bending vibrations of nitrate ions lying in this spectrum region (Figure 5a). 

The position and intensity of the transmission bands corresponding to vibrations of nitrate ions in these spectra are very close for all samples, and the intensity of the bands increases with an increase in the concentration of solutions. It should be noted that from the IR spectra of *Ln*(NO_3_)_3_ · *x*H_2_O solutions with low-intensity, overlapping, or even partially absent transmission bands, it is quite difficult to identify nitrate ions coordinated monodentately by *Ln*^3+^ ions. However, judging by the spectra, part (from ~10% to ~50%) of the nitrate ions is coordinated by *Ln*^3+^ ions mono- or bidentately (mainly monodentately), and the remaining nitrate groups are uncoordinated. 

The IR spectra of *Ln*(NO_3_)_3_ · *x*H_2_O salts contain transmission bands both observed for solutions (marked with * in Table S3 and in the text) and those belonging only to the salts. Thus, bidentately coordinated nitrate ions correspond to intense bands at ~1460 cm^−1^* and 1280 cm^−1^, medium-intensity bands at ~1660 cm^−1^* (in solutions, this band is present for Gd and Yb) (Figure 5d), and 1044 cm^−1^*. The difference between the band values at 1280 and 1044 cm^−1^ (Δ = 236 cm^−1^) indicates bidentate chelating of nitrate ions [61].

Therefore, the results of IR spectroscopy of commercial *Ln*(NO_3_)_3_ · *x*H_2_O salts with *Ln* = Ce, Sm, Gd, Yb confirm the structural analysis data known from the literature on the implementation of only bidentately coordinated nitrate groups in them. As for *Ln*(NO_3_)_3_ · *x*H_2_O solutions, according to IR spectroscopy, in dilute solutions of 20 mM there are *Ln*^3+^ ions coordinated by nitrate groups, but in very small quantities, bidentantly and monodentantly (mainly) linked with *Ln*^3+^ ions. The number of monodentate nitrate groups increases from Ce to Yb: the ratio of the intensity of the band (shoulder) at 1750 cm^−1^ to the intensity of the water bending vibration band at 1635 cm^−1^ increases from Ce to Yb (Figure 5e). 

It should be noted that the peculiarity of the diffraction patterns of *Ln*(NO_3_)_3_ · *x*H_2_O solutions (*Ln* = Ce, Sm, Gd, Yb) with c = 50 and 134 mM (Figure 4) is also preserved in the IR spectra of solutions with c = 20 and 50 mM (Figure 5b,d) and is consistent with the EXAFS data: an increase in the number of monodentate nitrate groups in the *Ln*^3+^ series with an increase in the degree of bond covalence.

The hydrolysis of *Ln*^3+^ cations is gaining increasing importance due to the growing interest in the biological aspects of their complexes [64,65,66]. In aqueous solutions of *Ln*(NO_3_)_3_ · *x*H_2_O salts, hydrolysis occurs—a chemical reaction between salt ions with H^+^ and OH^−^ ions of water. *Ln*(NO_3_)_3_ · *x*H_2_O salts are formed by the weak base of the multivalent *Ln*^3+^ ion and the strong HNO_3_ acid [67]; therefore, hydrolysis occurs through cations (pH < 7), which bind to the water anion in a stepwise manner (the general stages of hydrolysis of *Ln*^3+^ nitrates are presented by analogy with Fe^3+^ nitrate [68] due to the fact that there are no works in the literature on the systematization of the hydrolysis of rare-earth element nitrates):

Stage I:

*Ln*(NO_3_)_3_ + HOH ↔ *Ln*(OH)(NO_3_)_2_ + HNO_3_ (I.1)

*Ln*^3+^ + 3NO_3_^−^ + HOH ↔ *Ln*(OH)^2+^ + 2NO_3_^−^ + H^+^ + NO_3_^−^ (I.2) 

*Ln*^3+^ + HOH ↔ *Ln*(OH)^2+^ + H^+^ (I.3)

Stage II:

*Ln*(OH)(NO_3_)_2_ + HOH ↔ *Ln*(OH)_2_NO_3_ + HNO_3_ (II.1)

*Ln*(OH)^2+^ + 2NO_3_^−^ + HOH ↔ *Ln*(OH)_2_^+^ + NO_3_^−^ + H^+^ + NO_3_^−^ (II.2)

*Ln*(OH)^2+^ + HOH ↔ *Ln*(OH)_2_^+^ + H^+^ (II.3)

Stage III:

*Ln*(OH)_2_NO_3_ + HOH ↔ *Ln*(OH)_3_ + HNO_3_ (III.1)

*Ln*(OH)_2_^+^ + NO_3_^−^ + HOH ↔ *Ln(*OH)_3_ + H^+^ + NO_3_^−^ (III.2)

*Ln*(OH)_2_^+^ + HOH ↔ *Ln*(OH)_3_ + H^+^ (III.3)

According to [69], in the formation of Dy(OH)_3_, the final product of stage III of dysprosium nitrate hydrolysis (III.3) is preceded in a very narrow pH range~6.9–7.1 by basic dysprosium salts, in particular, Dy(NO_3_)(OH)_2_. The authors of [70] believe that the lanthanides are quite sensitive towards the water content of the medium and the aqueous ions *Ln* are hydrolyzed in water according to the equation [*Ln* + (H_2_O)_n_]^3+^ + H_2_O ↔ [*Ln*(OH)(H_2_O)_n−1_]^2+^ + H_3_O^+^. The authors then neglected the molecules of hydration water and presented the number, nature of the species in solution, and their hydrolysis constants for *Ln*^3+^ = Ce, Pr, Nd, Eu, Sm at c = 0.1 mol/dm^3^ (100 mM) and at 25 °C, which can be described as *Ln*^3+^ + HOH ↔ *Ln*(OH)^2+^ + H^+^ (I.3). 

Hydrolysis at stage II and, in particular, at stage III practically does not occur at room temperature, at which we conducted the experiment. This is also facilitated by ions that are formed during hydrolysis at stage I, which suppress hydrolysis at stage II, shifting the equilibrium of reactions to the left. In accordance with Le Chatelier’s principle, when diluting solutions (decreasing the concentration of hydrogen ions), the degree of hydrolysis increases. The tendency towards hydrolysis increases with an increase in atomic number and a decrease in ionic radius of *Ln* [70]: when moving from “light” to “heavy” *Ln*, hydrolysis increases. 

The degree of hydrolysis can be influenced by the composition of the substances involved in hydrolysis and the concentration of hydrolysis products, as well as the process temperature. The structure of *Ln*(NO_3_)_3_ in solutions depends on the degree of hydrolysis of solutions: the composition of the outer and inner spheres (the presence and content of nitrate ions and water molecules) and the density of ligands (the same groups). 

**Figure 6:**
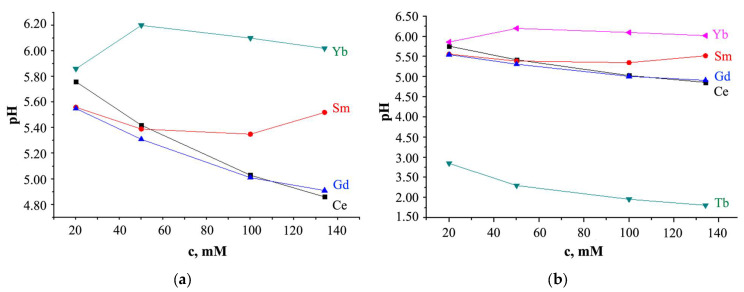
Relationship between the pH value and the concentration of *Ln*(NO_3_)_3_ · *x*H_2_O solution with (**a**) *x* = 6 for *Ln* = Ce, Sm, Gd and *x* is unknown for Yb; (**b**) *x* = 6 for *Ln* = Ce, Sm, Tb, Gd and *x* is unknown for Yb (pH measurement error is ±0.03).

For solutions of *Ln*(NO_3_)_3_ · *x*H_2_O salts (t = 25 °C; *Ln* = Ce, Sm, Tb, Gd, Yb), pH < 7, but the pH values are different (Figure 6). 

For *Ln* = Ce and Gd, with increasing solution concentration, the pH value decreases (Figure 6); for *Ln* = Sm, pH values first decrease from pH = 5.52 (c = 134 mM) to 5.35 (c = 100 mM), and then increase from pH = 5.39 (c = 50 mM) to 5.56 (c = 20 mM); at c > 20 mM, turbidity of the solution was observed; the pH value of a solution with Yb increases with an increase in its concentration with a maximum value of pH = 6.20 at c = 50 mM. 

As can be seen from Figure 6, the Tb(NO_3_)_3_ · *x*H_2_O salts solution is distinguished from other solutions by lower pH values, which smoothly decrease with increasing solution concentration. This behavior of the *Ln*(NO_3_)_3_ · *x*H_2_O solution with *Ln* = Tb, which differs from the behavior of solutions with *Ln* = Ce, Sm, Gd, Yb, is consistent with the EXAFS data (Figure 2) and with X-ray data (Figure 4d), which also stands out among other solutions. Different hydrolysis products, more or less complex, are formed depending on the type of *Ln* and anion, the solution concentration, and the pH value. So, according to [71], the *Ln*(OH)(NO_3_)_3_^−^ (in particular, *Ln* = La, Lu) anion complexes contain the *Ln*^3+^ ions, and the LnO(NO_3_)_3_^−^ (in particular, *Ln* = Ce) anion complexes contain the *Ln*^4+^ ions; for *Ln* = Pr and Tb it is proposed that the oxidation states are intermediate between *Ln*^3+^ and *Ln*^4+^. In solutions of *Ln* chlorides, *Ln*(H_2_O)_9_]^3+^ complexes with *Ln* = La, Pr, Nd [72], and *Ln*(H_2_O)_8_]^3+^ with *Ln* = Tb-Lu [73] were found, and in solutions of Tb sulfates, Tb(H_2_O)_6_]^3+^ complex was discovered [74]. The fewer water molecules in the immediate environment of *Ln* ions, the smaller the average *Ln*-O interatomic distance, the smaller the size of the *Ln* ion, the higher the hydrolysis rate, and the lower the pH [75]. Thus, it is not excluded that the smaller interatomic distance Tb-O in the nitrate solution (Figure 2) is due to the smaller number of water molecules in the inner sphere of the complex with terbium compared to other *Ln*, which contributes to a decrease in pH. 

It is necessary to pay attention to the obvious increase in pH with increasing temperature from 21 °C (the temperature at which the XAS solution experiments were performed) to 25 °C (the temperature at which IR spectra were obtained and the pH of the solutions was determined), which should be accompanied by an increase in hydrolysis. In the case of a salt formed with a weak base and a strong acid (our case), the *Ln*^3+^ cation undergoes hydrolysis, and the reaction is accompanied by the formation of H_3_O^+^ ions (their presence was not excluded when analyzing the IR spectra of solutions) (Table S3, Figure 5). An increase in the H_3_O^+^ content in the solution leads to a decrease in the concentration of OH^−^ ions. 

**Figure 7:**
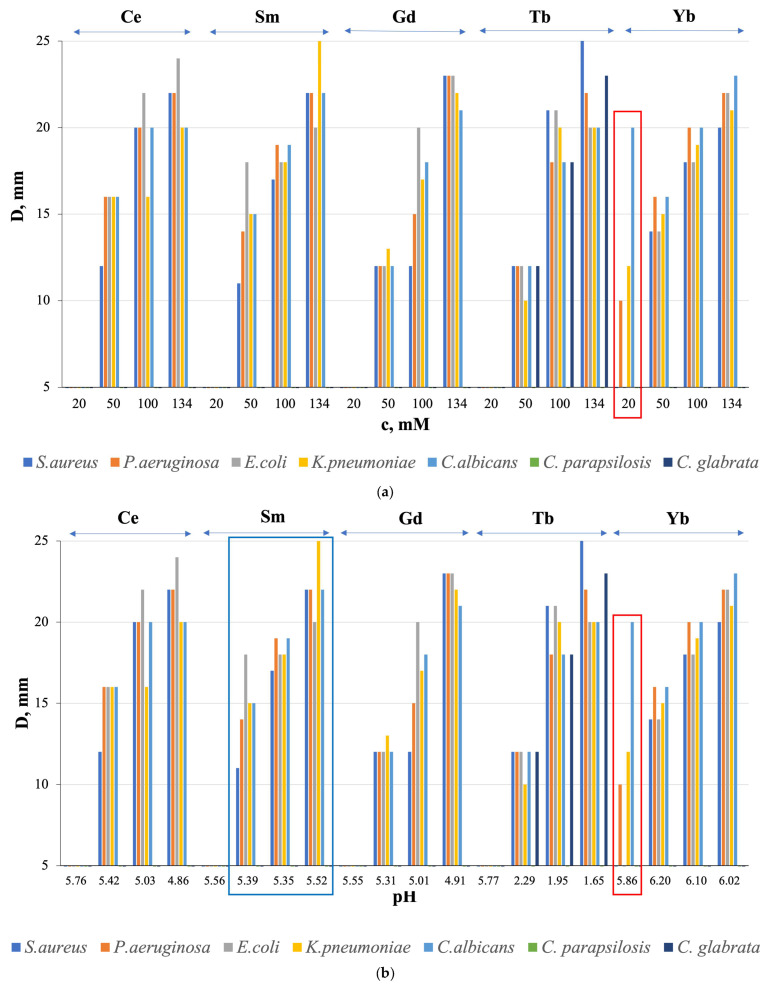
Relationship between the growth inhibition zone (D, mm) and (**a**) concentration (c, mM) and (**b**) pH of *Ln*(NO_3_)_3_ · *x*H_2_O solutions (*Ln* = Ce, Sm, Gd, Tb, Yb). Convergence of results based on three independent measurements; D ± 0.02 mm.

To establish the influence of the type of *Ln*, concentration and pH on the antimicrobial activity of *Ln*(NO_3_)_3_ · *x*H_2_O solutions, solutions with *Ln* = Ce, Sm, Gd, Tb, Yb from the beginning, middle, and end of the *RE* series, were selected. Figure 7 shows the relationship between the growth inhibition zone value (D, mm value) and the solution concentration (Figure 7a) and pH (Figure 7b), respectively.

All solutions with *Ln* = Ce, Sm, Gd, Tb, Yb showed average antibacterial activity at c = 50, 100, and 134 mM, except for *Ln* = Gd and Tb with c = 50 mM with low activity against all microorganisms, and *Ln* = Sm with c = 134 mM with high activity against *K. pneumoniae* (Figure 7a). Antifungal properties against the fungi *C. albicans* and *C. glabrata* were also found, with medium activity in all solutions with c = 50, 100, and 134 mM and with high activity for *Ln* = Tb with *c*= 100 and 134 mM (Figure 7a). Moreover, antimicrobial activity is absent for solutions with a concentration of *c* = 20 mM, except for a solution with *Ln* = Yb (Figure 7a, red rectangle). At the same time, the sensitivity of the fungus *C. albicans* to a solution with *c* = 20 mM is higher than to a solution with c = 50 mM. We detected the observed “homeopathic” effect for the first time for these objects.

The microorganism growth inhibition zone (D, mm value) has a general tendency to increase with increasing solution concentration (Figure 7a) with the best results at c = 134 mM for *Ln* = Sm and Tb, respectively, in relation to the bacterium *K. pneumoniae* and the fungi *C. albicans* and *C. glabrata* (Figure 7b).

HNO_3_ solutions do not exhibit AMA (except for HNO_3_ with pH = 1.98 and with D = 18 mm in relation to *S. aureus*), which indicates a connection between biocidal properties and *Ln*^3+^ ions, and not with NO_3_^1−^ ions, and the absence of a connection with the pH (hydrogen index) of HNO_3_. As for the relationship between the value of D, mm and the pH of *Ln*(NO_3_)_3_ · *x*H_2_O solutions, the general tendency of increasing D, mm with decreasing pH was found for *Ln* = Ce, Gd, Tb (the maximum value of the growth inhibition zone of the fungus *C. glabrata* was achieved at pH = 1.65), and Yb (in the pH range from 6.20 to 6.02) (Figure 7b); for *Ln* = Sm, the opposite effect was obtained (Figure 7b) with the maximum growth inhibition zone of *K. pneumoniae* bacteria at pH = 5.52, and for *Ln* = Yb at pH = 5.86 the values of D, mm are less than for pH = 6.02 (Figure 7b). It follows that, in general, there is no relationship between AMA and solution pH.

Judging by the obtained results, the antimicrobial activity of the studied *Ln*(NO_3_)_3_ · *x*H_2_O solutions is influenced by the phase purity of the samples and the nature of *Ln*, which determines the composition and structure of nitrates and, in some cases, the pH of the solution, which in turn is associated with its concentration and vice versa. So, from the entire *Ln* series studied, a solution with *Ln* = Yb (non-single-phase sample) (Figure 2, red arrow) and with *Ln* = Tb (Figure 2) stands out. On the other hand, according to [5], *Ln* have similar ionic radii to calcium but, due to their higher charge (*Ln*^3+^), *Ln* ions have a high affinity for Ca^2+^ sites on biological molecules, and therefore *Ln*^3+^ are able to block calcium channels. Thus, even though the Ln^3+^ ions themselves cannot cross cell membranes, they can act by blocking the exterior face of the calcium channel. It is possible that the absence of direct quantitative or semi-quantitative correlations with individual characteristics of *Ln*(NO_3_)_3_ · *x*H_2_O solutions is due to the different composition and structure of bacteria and fungi, as well as individual bacteria, making these relationships less pronounced.

Compounds with rare-earth ions are biocompatible, relatively cheap, and are included as additives in anti-inflammatory, regenerating, analgesic, wound-healing, and antimicrobial ointments and preparations, such as EPLAN, which contains lanthanum nitrate [76]. First obtained data on the antibactericidal and antifungicidal (unique results) activity of solutions of commercial *Ln*(NO_3_)_3_ · *x*H_2_O salts with *Ln* = Ce, Sm, Gd, Tb, Yb, including the manifestation of biocidal properties of Yb(NO_3_)_3_·*x*H_2_O solution with c = 20 mM, make it possible to consider these objects as potential antimicrobial drugs, expanding the range of known materials.

One should not forget about bacteria and fungi, the composition and structure of which must be taken into account when considering the antimicrobial process, which is beyond the scope of this work.

## 3. Materials and Methods

The nitrate salts *Ln*(NO_3_)_3_ · *x*H_2_O with 99.9% *Ln*= Ce(x = 6), Pr, Nd(x = 6), Sm(x = 6), Eu, Gd, Tb(x = 6), Dy(x = 5), Ho(x = 5), Er, Tm, Yb, Lu were purchased from LANHIT LTD (Russia) and used as received. We studied 20 (in most measurements), 50, 100, and 134 mM *Ln*(NO_3_)_3_ · *x*H_2_O aqueous solutions (separate measurements were performed for solutions with *Ln* = Ce, Sm, Gd, Yb) with water pH 6.2. All solutions were prepared using ultrapure water (Milli-Q Advantage A10 Water Purification System, Millipore, France).

### 3.1. X-ray Studies

X-ray experiments have been carried out at the LANGMUIR beamline (Kurchatov Center for Synchrotron Radiation, Russia) [77]. The Langmuir trough was filed with water vapor-saturated helium to decrease X-ray scattering. Aqueous solutions of *Ln*(NO_3_)_3_ · *x*H_2_O salts at a concentration of 20 mM were filled in the Langmuir trough mounted on the diffractometer. All measurements were performed at room temperature, t = 21 °C.

#### 3.1.1. X-ray Absorption Spectroscopy Measurements at Liquid Surface

*Ln*(NO_3_)_3_ aqueous solutions were measured in fluorescence mode under TER geometry. The channel-cut monochromator Si (111) with spectral width of ~2 eV was used to perform energy scan over the range of 400 eV. *Ln*^3+^ L3-edge absorption spectra were collected at the fixed incidence angle of 0.9 × θC (θC is the critical angle of TER for water). The energy dispersive Vortex EX detector was mounted above the water subphase at an angle of 90°. To obtain high-quality EXAFS spectra, we averaged several energy scans recorded in the multipass mode of data acquisition. The reproducibility of the energy position of the monochromator was determined to be within 0.18 eV.

#### 3.1.2. X-ray Absorption Spectroscopy Measurements of Powder Samples

*Ln*(NO_3_)_3_ · *x*H_2_O microcrystalline samples were studied at the Structural Materials Science beamline (Kurchatov Center for Synchrotron Radiation, Moscow, Russia) [78]. EXAFS spectra at the lanthanide L3-edge were collected in transmission mode using two ionization chambers filled with N_2_/Ar mixtures.

#### 3.1.3. XRSW Measurements

The fluorescence intensity was recorded by an energy dispersive Vortex EX detector (Hitachi, Japan) mounted above the water subphase at an angle of 90°. The characteristic fluorescence spectra were collected for each angle of incidence in the angular range corresponding to the TER region.

### 3.2. EXAFS Data Analysis

The extraction of the experimental fine-structure χ(k) from the atomic background function was performed using conventional procedures described elsewhere [79,80]. EXAFS data analysis was carried out in the single-scattering approximation based on the equation, which describes EXAFS as a sum of the contributions from different coordination shells with radii Ri and coordination numbers Ni:

$$\chi \left(k\right)=S_{0}^{2}\sum_{i=1}^{n}\frac{N_{i}F_{i}\left(k\right)}{R_{i}^{2}k}e^{\frac{-2R_{i}}{\lambda \left(k\right)}}e^{-2\sigma_{i}^{2}k^{2}}sin\left(2kR_{i}+\psi_{i}\left(k\right)\right)$$

where $S_{0}^{2}$ is a many-body reduction factor, that accounts for amplitude damping due to the multielectron effect; $F_{i}\left(k\right)$ and $\psi_{i}\left(k\right)$ are the backscattering amplitude and phase shift for photoelectron scattered by neighbor atoms; λ(k) is the mean-free-path of the photoelectron; $\sigma_{i}^{2}$ is Debye–Waller factor, which represents the mean square displacement of an atom from equilibrium position. Backscattering amplitude $F_{i}\left(k\right)$ and phase shift $\psi_{i}\left(k\right)$ were calculated using the ab initio code FEFF8.5L [81,82]. The photoelectron inelastic losses were accounted for within the one-plasmon approximation using the complex exchange-correlation Hedin−Lundqvist potential [83]. FEEF calculations were performed for atomic clusters, which include the nearest environment of lanthanide ions: 4 H_2_O molecules and 3 NO_3_ ligands. Such calculations allow performing EXAFS spectra fitting using paths associated with H_2_O and NO_3_ molecules. Fourier transformation is calculated for χ(k) × k^2^ using k-range = 2–9 Å^−1^; k^2^-weighted EXAFS data were fitted in space of their Fourier transforms |FT(χ(k) × k^2^)|. The value of $S_{0}^{2}$ factor was fixed at 0.9. All data processing and quantitative analysis of EXAFS spectra were performed using Larch 0.9.80 software package [84].

EXAFS spectra obtained in our experiments for Ce and Pr exhibited distinct signs of multi-electron excitation, which is a well-known feature of the electronic structure of these ions [85]. Thus, in analysis of the EXAFS spectra for Ce and Pr we used the ATHENA program from the IFFEFIT package [86] to exclude the influence of multi-electron excitation effect.

### 3.3. X-ray Diffraction Experiments

X-ray diffraction experiments with *Ln*(NO_3_)_3_ · *x*H_2_O aqueous solutions of the beginning (Ce and Sm), middle (Gd) and end (Yb), ions of the *Ln* series with concentrations of c = 50 and 134 mM were tested using an Al container and difractometer PowDiX 600 (ADANI, Minsk, Belarus) equipped with a MYTHEN2 R 1D (Dectris) detector at room temperature (t = 21 °C), using monochromatic CuKα1 radiation (λ = 1.5406 Å) with a Ni-filter 0.02 mm thick, on a diffracted beam; θ-2θ, 2θ ± 0.01°), and continuous shooting in the range of 5–60° (30 kV, 10 mA).

### 3.4. FT-IR Spectroscopy

Fourier-transform infrared spectra of *Ln*(NO_3_)_3_ · *x*H_2_O (*Ln* = Ce, Sm, Gd, Yb) solutions (c = 20 and 50 for *Ln* = Ce, Sm, Gd, Yb; c = 100 for *Ln* = Yb) were collected in a FSM-2201 (Infraspec, Saint Petersburg, Russia) spectrometer in the 500–4000 cm^−1^ range. An average of 15 scans per sample with a resolution of 1 cm^−1^ formed each spectrum. Aqueous solutions were analyzed using an ZnSe Attenuated Total Reflectance (ATR) accessory. A Fourier-transform infrared spectrometer WQF-530A (BFRL, Beijing, China) was used to study *Ln*(NO_3_)_3_ · *x*H_2_O (*Ln* = Ce, Sm, Gd, Yb) salts: wavenumber range 500–4000 cm^−1^, spectral resolution 0.85 cm^−1^, number of scans 256.

### 3.5. The Antimicrobial Activity

The study of antimicrobial activity (AMA) of *Ln*(NO_3_)_3_ · *x*H_2_O (*Ln* = Ce, Sm, Gd, Tb, Yb) solutions (*c* = 20, 50, 100, 134 mM) was carried out by the disk diffusion method for antimicrobial activity against bacteria (*S. aureus*, *E. coli*, *P. aeruginosa*, *K. pneumoniae*) and fungi (*C. albicans*, *C. glabrata*, *C. parapsilosis*). The test bacterium was seeded as a “lawn” in Petri dishes on Mueller–Hinton agar. A thin-walled cylinder (6–8 mm in diameter) was used to make holes on the agar surface, into which test samples were added in the powder form with the addition of 2–3 drops of NaCl physiological solution (0.9 wt.%). Next, Petri dishes were placed in a thermostat at 37 °C for 24 h. The sensitivity of microorganisms to the objects under study was assessed by the bacterial growth inhibition zone (D, mm) around the hole (including the hole diameter) using a ruler [87]. The sensitivity degree of microorganisms to the studied samples depends on the D, mm value: the larger it is, the higher the sensitivity. According to the classification presented in [88], there are four groups of growth inhibition zone diameters of microorganisms in the presence of antimicrobial agents: D < 10 mm indicates a lack of sensitivity, D = 11–15 mm indicates low sensitivity, D = 15–25 mm denotes an average sensitivity, and D > 25 mm represents high sensitivity to the drug. Microbiological studies were carried out in a second-class microbiological protection box, equipped with a UV lamp and laminar air flow.

## 4. Conclusions

Based on the analysis of the results of studying commercial *Ln*(NO_3_)_3_ · *x*H_2_O salts (X-ray diffraction, IR spectroscopy, XAS) and their diluted solutions (XAS), as well as solutions with *Ln* = Ce, Sm, Gd, Yb (X-ray diffraction, pH measurement, IR spectroscopy) together with literature data, it was shown that in solution, a few nitrate anions can be coordinated bidentantly and monodentantly depending on the type of *Ln*, and also be uncoordinated. Water molecules are chemically bonded to *Ln* ions via oxygen and, in addition, form multiple hydrogen bonds with water molecules and nitrate groups.

For the first time, X-ray absorption spectroscopy under total external reflection geometry has been used to study the local environment of lanthanide ions in dilute aqueous solutions (20 mM *Ln*(NO_3_)_3_ · *x*H_2_O). The applied experimental technique allowed for high-quality EXAFS data to be collected for all elements from Ce to Lu (except for Pm). All solutions were measured under identical experimental conditions, all EXAFS spectra were processed using the same software package and modeling procedures, which provided significant advantages in comparative studies of lanthanide coordination. A comparison of *Ln*-O interatomic distances for salts and their solutions, determined by the EXAFS method, showed that for a number of lanthanides from the middle of the lanthanide series, the oxygen coordination sphere contracts in solutions, which may be due to partial or complete dissociation of the nitrate complex in water.

The obtained data for *Ln*(NO_3_)_3_ · *x*H_2_O salts with specific compositions and structures, and their solutions, showed the need to characterize the initial samples synthesized by researchers or commercially available. Only salts and their solutions being identical in composition and structure makes it possible to correctly analyze the conducted research. Otherwise, the discrepancy between the results of an experimental study regarding “the same objects” or calculations within the experiment may lead to misunderstandings.

The revealed biocidal properties of solutions of commercial *Ln*(NO_3_)_3_ · *x*H_2_O) salts with *Ln* = Ce, Sm, Gd, Tb, Yb make it possible to continue studying the antimicrobial activity of both *Ln*(NO_3_)_3_ · *x*H_2_O salts and solutions with their characterization by different methods for the entire *Ln* series. This will allow to establish correlations between the characteristics of drugs with antimicrobial properties and the composition and structure of microorganisms to identify optimal combinations for creating new medical objects.

## Data Availability

Data available on request.

## Supplementary Materials

The following supporting information can be downloaded at: https://www.mdpi.com/article/10.3390/molecules29174023/s1, Table S1: structural parameters derived from EXAFS fitting procedure; Table S2: average Ln-O interatomic distances in the first coordination sphere in the structures of *Ln*(NO_3_)_3_ · *x*H_2_O salts; Table S3: assignment of the bands in the IR spectra of the *Ln*(NO_3_)_3_ · *x*H_2_O aqueous solutions; Figure S1: Fourier-transformed EXAFS spectra for *Ln*(NO_3_)_3_ · *x*H_2_O salts. Experimental spectra are shown using black color, and red color is used to plot fitted curved; Figure S2: Fourier-transformed EXAFS spectra for *Ln*(NO_3_)_3_ · *x*H_2_O aqueous solutions. Experimental spectra are shown using black color, and red color is used to plot fitted curved; Figure S3: the experimental and calculated EXAFS-signals χ(k)*k^2^ for *Ln*(NO_3_)_3_ · *x*H_2_O aqueous solutions. Experimental spectra are shown using black color, and red color is used to plot fitted curved; Figure S4: the experimental and calculated EXAFS-signals χ(k)*k^2^ for *Ln*(NO_3_)_3_ · *x*H_2_O crystalline powders. Experimental spectra are shown using black color, and red color is used to plot fitted curved; Figure S5: *Ln* L_III_ XANES spectra for *Ln*(NO_3_)_3_ · *x*H_2_O salts (black color) and aqueous solutions (red color). Vertical lines are used to mark the position of the second maximum.
