# Supplementary material for: Physicochemical Characterization and Antimicrobial Properties of Lanthanide Nitrates in Dilute Aqueous Solutions
Source: Molecules. 2024 Aug 25;29(17):4023. doi: 10.3390/molecules29174023 (PMC11396220; doi:10.3390/molecules29174023)
Supplement: Supplementary file 1 [file molecules-29-04023-s001.zip › molecules-3104847-supplementary.pdf]

molecules

MDPI

Supplementary Materials

Table S1. Structural parameters derived from EXAFS fitting procedure.

***Ln* type**	***Ln*-O**			***Ln*-N**			**ΔE, eV**
	**CN**	**R, Å**	**σ^2*10^3, Å^2**	**CN**	**R, Å**	**σ^2*10^3, Å^2**	
			Ln*(NO$_3$)$_3$ · *x*H$_2$O aqueous solutions with nitrate groups and water molecules in the local environment of *Ln				
Ce	9.7±1.4	2.53±0.04	9.6±1.8	2.6±1.2	3.20±0.04	**3**	3.13
Pr	10±4.3	2.51±0.02	10±2	2.1±1.3	3.16±0.05	**5**	-2.6
Nd	10.7±5.1	2.49±0.05	10	2.2±3.7	3.11±0.13	**4**	-3.9
Sm	10.9±1.0	2.47±0.01	8.7±1.6	1.2±0.9	3.12±0.06	**4**	-2.4
Eu	12.9±4.6	2.47±0.03	10.7±4.9	1.5±2.9	2.91±0.15	**6.4**	-1.7
Gd	9.8±2.3	2.42±0.02	9.3±3.3	0.7±2.5	2.99±0.26	**8.2**	-2.2
Tb	11.4±3.7	2.35±0.02	14±1	1.3±1.8	2.94±0.04	**10**	-6.2
Dy	10.2±5.2	2.37±0.03	10.2±2.3	0.8±2.1	2.97±0.32	**6**	-3.6
Ho	12.2±5.4	2.34±0.04	11.1±4.4	1.1±3.7	2.99±0.21	**4**	-4.9
Er	11.6±2.9	2.35±0.02	12.2±3.2	0	-	**-**	-3.6
Tm	12.1±4.4	2.34±0.03	10.5±4.4	0	-	**-**	-2.9
Yb	11.7±3.4	2.33±0.03	8.8±2.0	0.9±2.9	2.91±0.02	**6.2**	-1.4
Lu	11.6±1.2	2.31±0.01	9.7±1.5	0	-	-	0.2
				***Ln*(NO$_3$)$_3$ · xH$_2$O salts**			
Ce	**10**	2.55±0.03	10±2.1	**3**	3.18±0.08	3±8	2.5
Pr	**10**	2.51±0.04	11±2	**3**	3.11±0.07	5±9	-3.2
Nd	**10**	2.51±0.03	9.2±1.5	**3**	3.06±0.05	4±7	-4.5
Sm	**10**	2.46±0.03	9.5±1.4	**3**	3.03±0.05	4±5.7	-5.5
Eu	**10**	2.47±0.01	10.6±0.5	**3**	3.05±0.02	6.4±2.8	-3.3
Gd	**10**	2.44±0.02	10.5±0.9	**3**	3.03±0.04	8.2±5.7	-2.9
Tb	**10**	2.42±0.02	10.6±0.9	**3**	3.01±0.04	10.0±7	-4.1
Dy	**10**	2.40±0.02	9.6±1.1	**3**	2.96±0.04	6.0±6.2	-5.5
Ho	**10**	2.39±0.01	9.4±0.9	**3**	2.97±0.03	4.0±3.7	-5.3
Er	**10**	2.39±0.02	9.6±0.9	**3**	2.96±0.03	3.0±3.6	-4.6
Tm	**10**	2.38±0.02	9.7±1.1	**3**	2.96±0.04	5.2±5.5	-3.1

Yb	**10**	2.30±0.02	9±0.9	**3**	2.85±0.04	6.2±6.0	-4.6
Lu	**10**	2.34±0.01	9.9±0.8	**3**	2.85±0.04	10.0±2.1	-1.3

Table S2. Average Ln-O interatomic distances in the first coordination sphere in the structures of *Ln*$(NO_3)_3 \cdot xH_2O$ salts.

		Ce	Pr	Nd	Sm	Eu	Gd	Tb	Dy	Ho	Er	Tm	Yb	Lu
**Ln-O**	R,Å	2.661	2.638	2.552	2.516	2.508	2.497	2.484	2.458/2.461*	2.446/2.449*	2.443/2.443*	2.438	2.425**	2.356/2.363***
**CN_O**	CN	11	10	10	10	10	10	10	10	10	10	10	10*	9/9***

*according to our samples; **for x=5; ***for x=4/x=3

Table S3. Assignment of the bands in the IR spectra of the *Ln*$(NO_3)_3 \cdot xH_2O$ aqueous solutions.

**Wavenumber, cm^{-1}**		**Assignment**	**References**
~1044*	1044	Due to bound nitrate	[18]
very weak	1030-1050	$\nu_{s(1)}(NO_3)$ - symmetric stretching of coordinated nitrate ion	[18]
	1041 (for Ho)	$\delta(NO_3)$	[57]
~1146	1135	$\delta(OH)$ – bending vibration	[61]
(~1170 for Gd and Yb)	1134	$\nu_2\ (H_3O)^+\ (NO_3)^-$	[59]
very weak			
~1345	1340	$\nu_{as(3)}(NO_3)$ - asymmetric stretching of solvated (non-coordinated) nitrate ion	[18]
middle	(for La)		
~1400	1416	$\nu_{as(3)}(NO_3)$ - asymmetric stretching of solvated (non-coordinated) nitrate ion	[18]
shoulder to ~1345			
~1460	1464-1467	$\nu_{as(1)}$ - the coordinated nitrate groups (C_{2v})	[60]
very weak			
~1470*	1455 (for La)	$\nu_{as(3)}(NO_3)$ - asymmetric stretching of mono- and bidentantly coordinated nitrate ion	[57, 60]
very weak		$\delta(NO_3)$	
	1482 (for Ho)	$\nu_{as(3)}(NO_3)$ - asymmetric stretching of mono- and bidentantly coordinated nitrate ion	[57]
	1464-1467		[18]
~1550	1540-1545 (for Ce)	ν_3–monodentate nitrate	[62]
very weak	1585-1550 (for Ce)	ν_3–bidentate chelating nitrate	
~1635*	1630	Bending mode of H_2O	[19]
strong, sharp	1640	$\delta(H_2O)$, $\nu(N{=}O)$	[57]
	1633 (for Ho)	δ(OH of H_2O)	[57]
~1750	1770-1730	$\nu_s(NO_3^-)+\delta_{ip}(NO_3^-)$	[19]
very weak, shoulder to ~1635			
~2114	2114	$\delta(H_2O)+\nu_L(H_2O)$	[63]
weak, wide	2130	$\nu_2 + \nu_L$ - the combination of oscillation $\nu_2 + \nu_L$: deformation together with libration	[63]
~3314 strong, wide	3315	$\nu(H_2O)$	[63]

molecules

MDPI

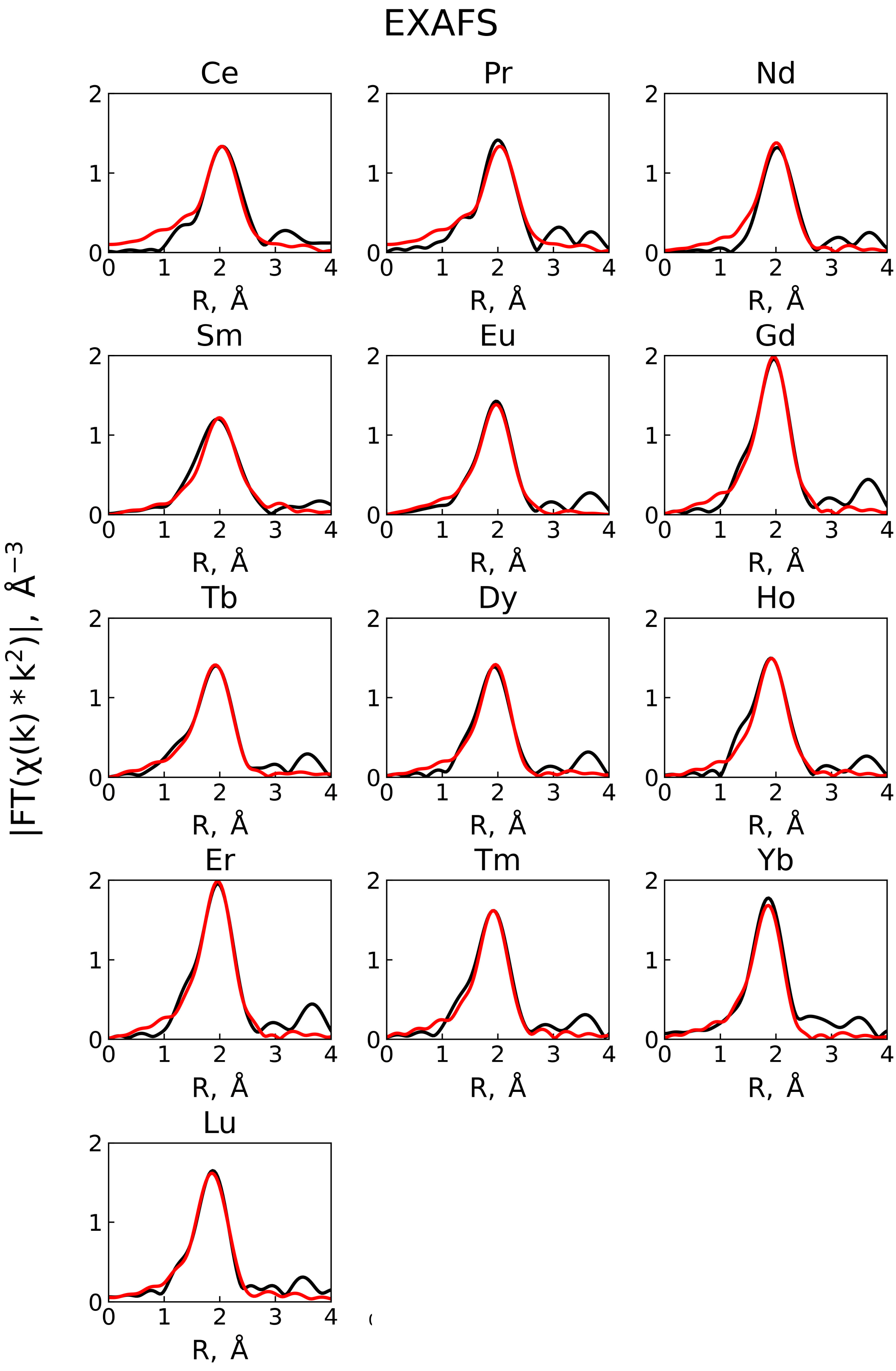

Figure S1. Fourier-transformed EXAFS spectra for *Ln*$(NO_3)_3 \cdot xH_2O$ salts. Experimental spectra are shown using black color, and red color is used to plot fitted curved.

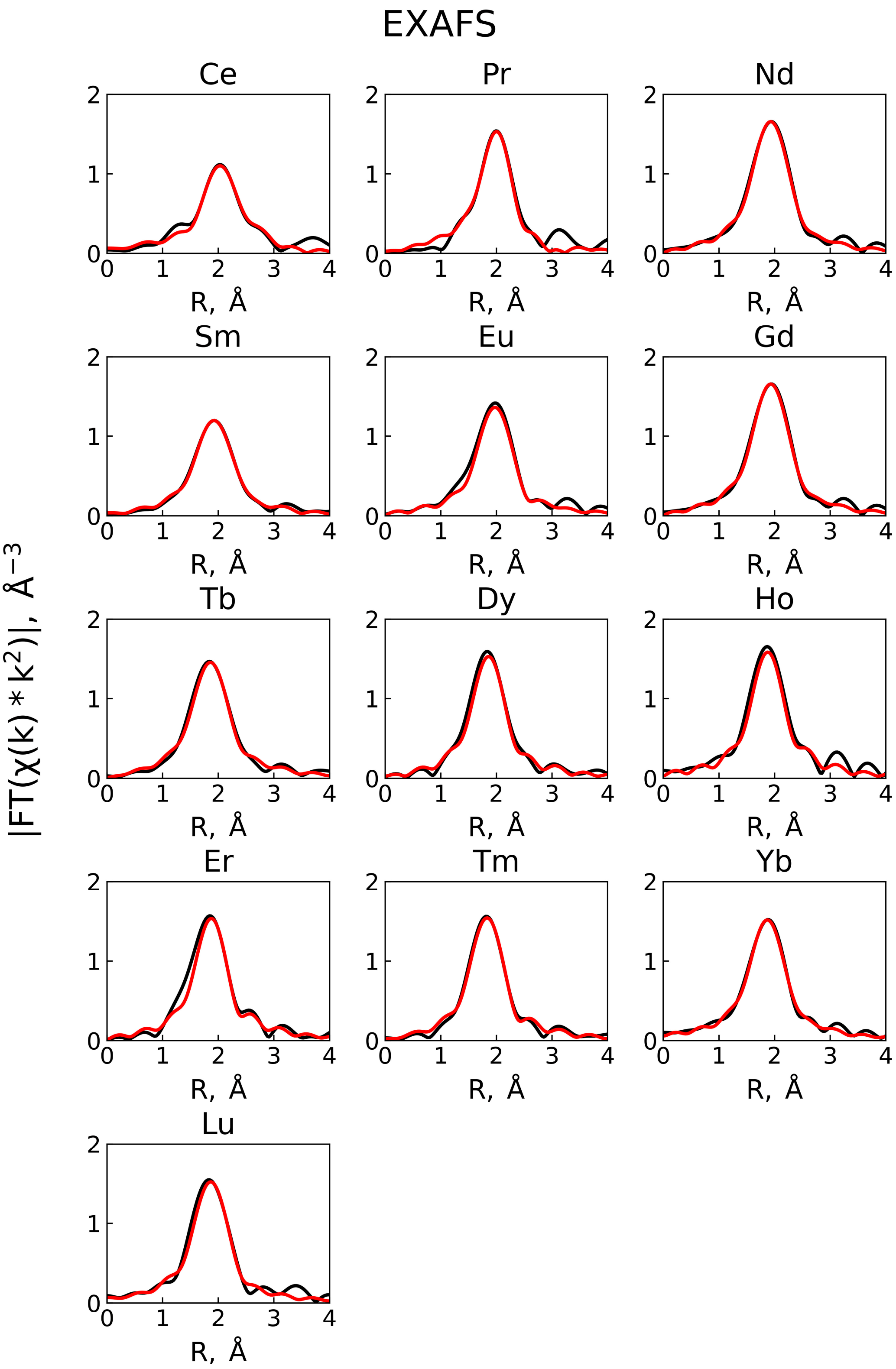

Figure S2. Fourier-transformed EXAFS spectra for *Ln*$(NO_3)_3 \cdot xH_2O$ aqueous solutions. Experimental spectra are shown using black color, and red color is used to plot fitted curved.

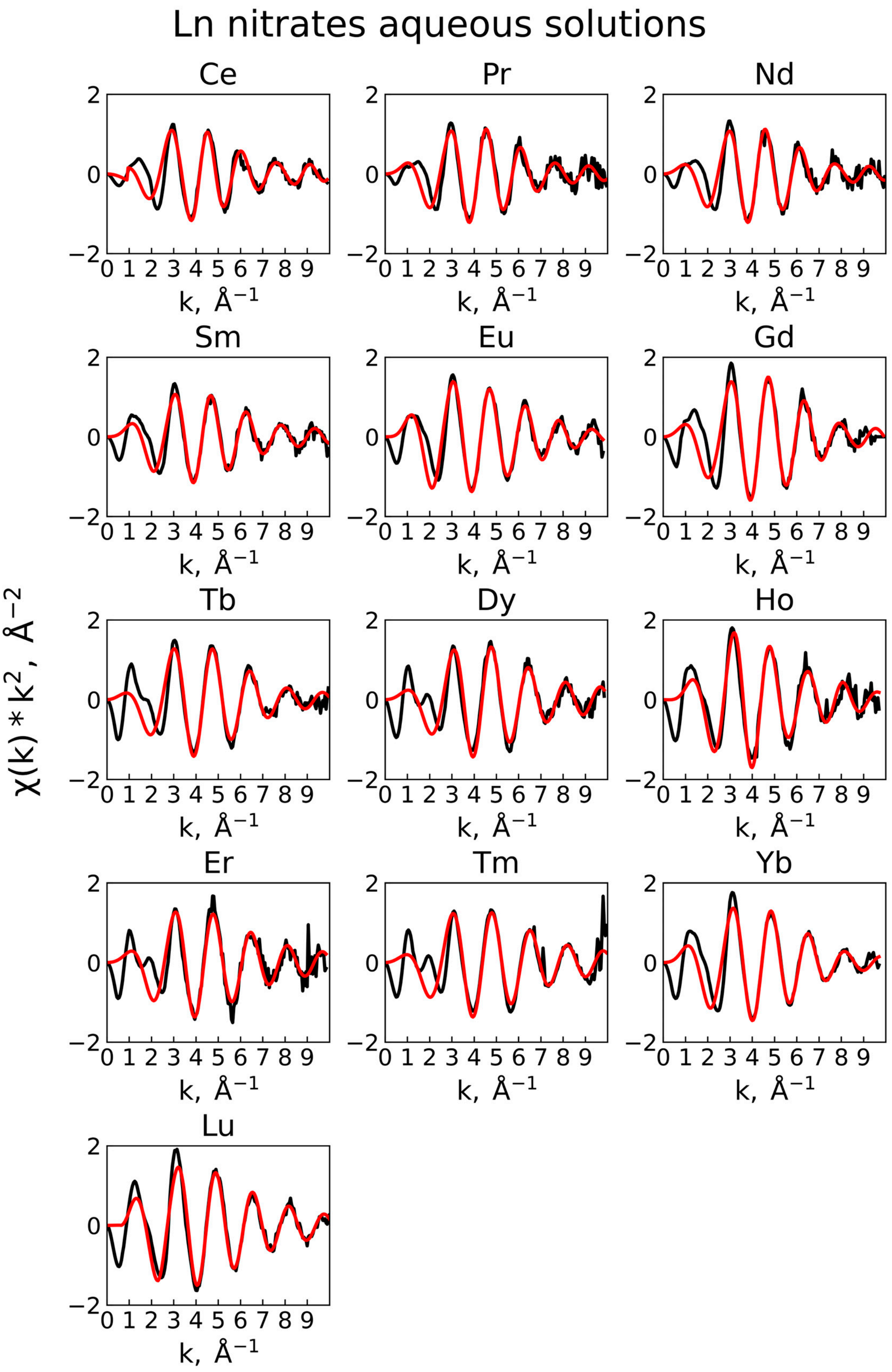

Figure S3. The experimental and calculated EXAFS-signals χ(k)*k^2 for $Ln(NO_3)_3 \cdot xH_2O$ aqueous solutions. Experimental spectra are shown using black color, and red color is used to plot fitted curved.

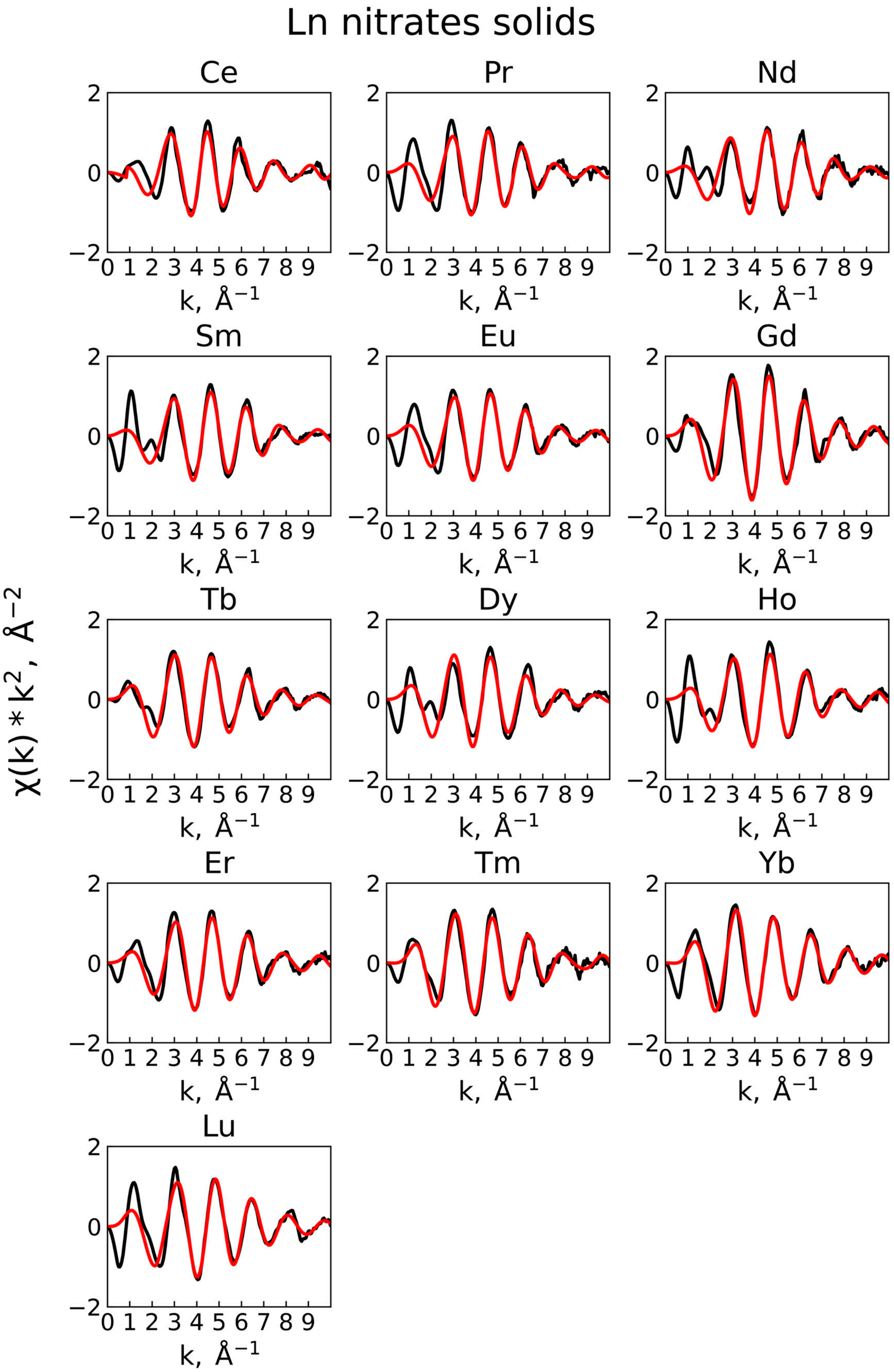

Figure S4. The experimental and calculated EXAFS-signals $\chi(k)*k^2$ for $Ln(NO_3)_3 \cdot xH_2O$ crystalline powders. Experimental spectra are shown using black color, and red color is used to plot fitted curved.

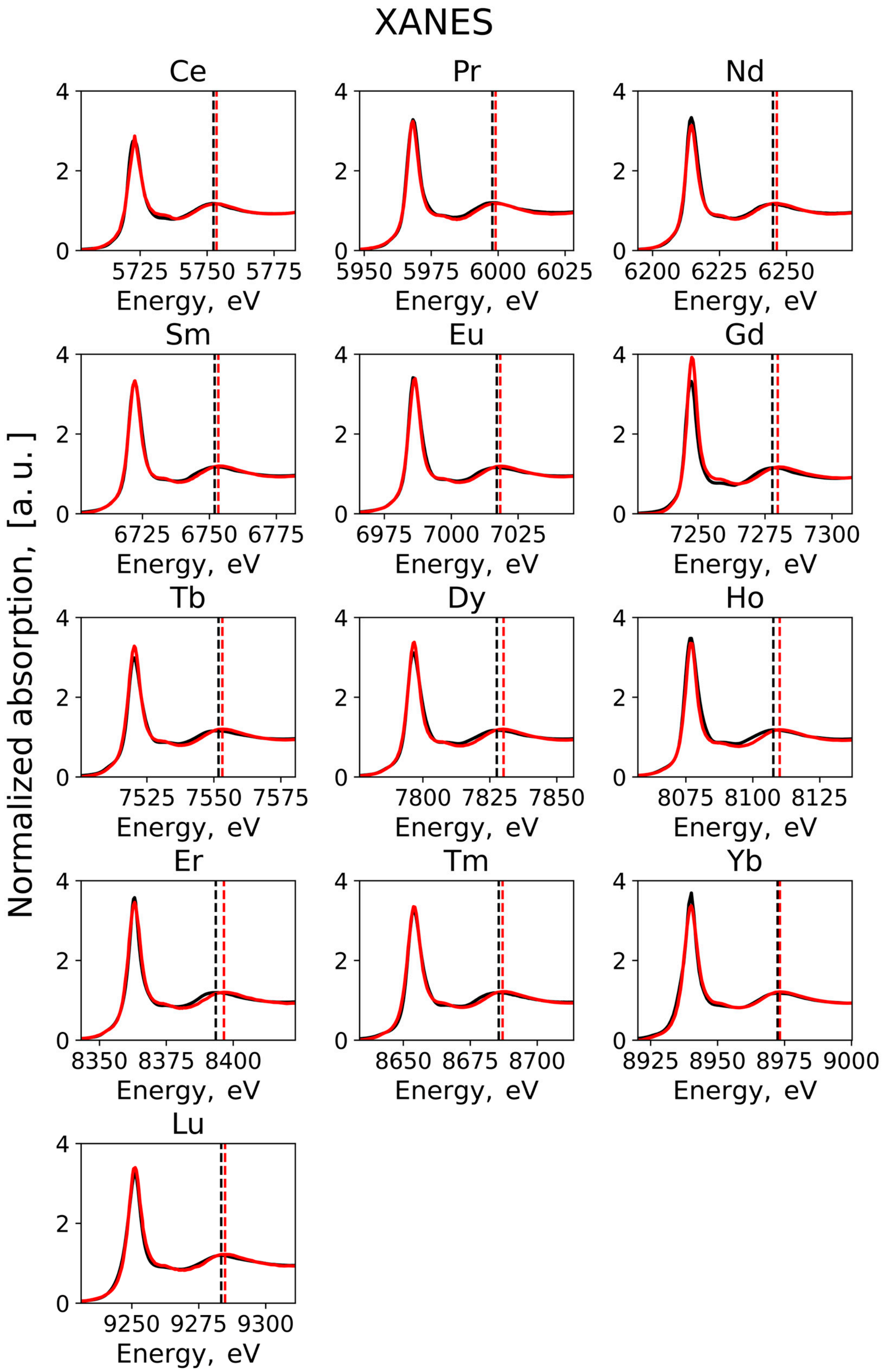

Figure S5. *Ln* LIII XANES spectra for *Ln*(NO_3)$_3$ · $x$$H_2O$ salts (black color) and aqueous solutions (red color). Vertical lines are used to mark the position of the second maximum.
